# Tislelizumab combined with anlotinib in the second-line treatment of malignant pleural mesothelioma

**DOI:** 10.1097/MD.0000000000032459

**Published:** 2022-12-30

**Authors:** Dandan Zhang, Jianping Liang, Yanhua Lv, Xikun Huang, Weihong Guo

**Affiliations:** a Zhongshan City People’s Hostipial, Zhongshan, China.

**Keywords:** anlotinib, immune checkpoint inhibitors, anti-angiogenic medication, malignant pleural mesothelioma, tislelizumab

## Abstract

Malignant pleural mesothelioma (MPM) is a malevolent tumor originated from pleura and often leads to poor prognosis. Chemotherapy of pemetrexed and cisplatin combined with antiangiogenic therapy of bevacizumab is recommended as the first-line regimen by guidelines. However, there are few sustainable second-line anti-tumor theraies that bring distinct survival benefit after the occurrence of drug resistance as the reported mPFS (median progression-free survival) scarcely exceeds 6 months. Immune checkpoint inhibitors are extensively investigated in pan-cancer, and dual immunotherapy has been listed in the first-line recommendation of MPM in several guidelines, while MPM patients benefit modestly from immune checkpoint inhibitors combination or monotherapy in second-line practice.

**Patient concerns and diagnosis:**

We report a 59-year-old male patient who was diagnosed with unresectable MPM in April 2021.

**Interventions:**

He received firstly pemetrexed combined with platinum and bevacizumab, which barely curbed disease progression; When the first line treatment failed, he was switched to tislelizumab combined with anlotinib.

**Outcomes:**

Tislelizumab combined with anlotinib significantly relieved his clinical symptoms, and imaging examination further validated the improvement. Until present, the second-line treatment PFS is more than 10 months.

**Lessons:**

The case firstly demonstrated the efficacy of tislelizumab combined with anlotinib in the second-line management of MPM. Thus, immunotherapy combined with small-molecule multi-target anti-angiogenic medication may be alternative for the second-line schemes of MPM.

## 1. Introduction

Malignant pleural mesothelioma (MPM) is a malevolent tumor originated from pleura and often leads to poor prognosis with 5-year overall survival (OS) rate of 5% to 10% for all stages. The first-line treatment relies on chemotherapy including pemetrexed and cisplatin, and combination of bevacizumab can prolong patient survival; however, few second-line anti-tumor strategies distinctly bring distinct survival benefit to resistant cases with progression-free survival (mPFS) less than 6 months. Immune checkpoint inhibitors (CKIs) are extensively investigated in pan-cancer, and dual immunotherapy has been listed for the first-line use of MPM in some international guidelines, while MPM patients benefit modestly from CKIs combination or monotherapy in second-line use. Basic researches and clinical practices of other tumor types have revealed that anti-angiogenic therapy such as anti-VEGF and multi-targeted small molecule anti- angiogenesis drugs can synergistically enhance the efficacy of immunotherapy by modulating tumor microenvironment, while little is reported on their combined use in MPM. Here we report a 59-year-old male patient who was diagnosed with unresectable malignant pleural mesothelioma in April 2021. The disease continued to progress under the first-line treatments of pemetrexed and platinum in combination with bevacizumab. Fortunately, the case responded favorably to second-line therapy, namely the combination of tislelizumab (PD-1 inhibitor) and anlotinib (small molecule multi-targeted anti-angiogenic drug). This report provides a new management paradigm for second-line treatment of MPM.

## 2. Case presentation

**Figure 1.**
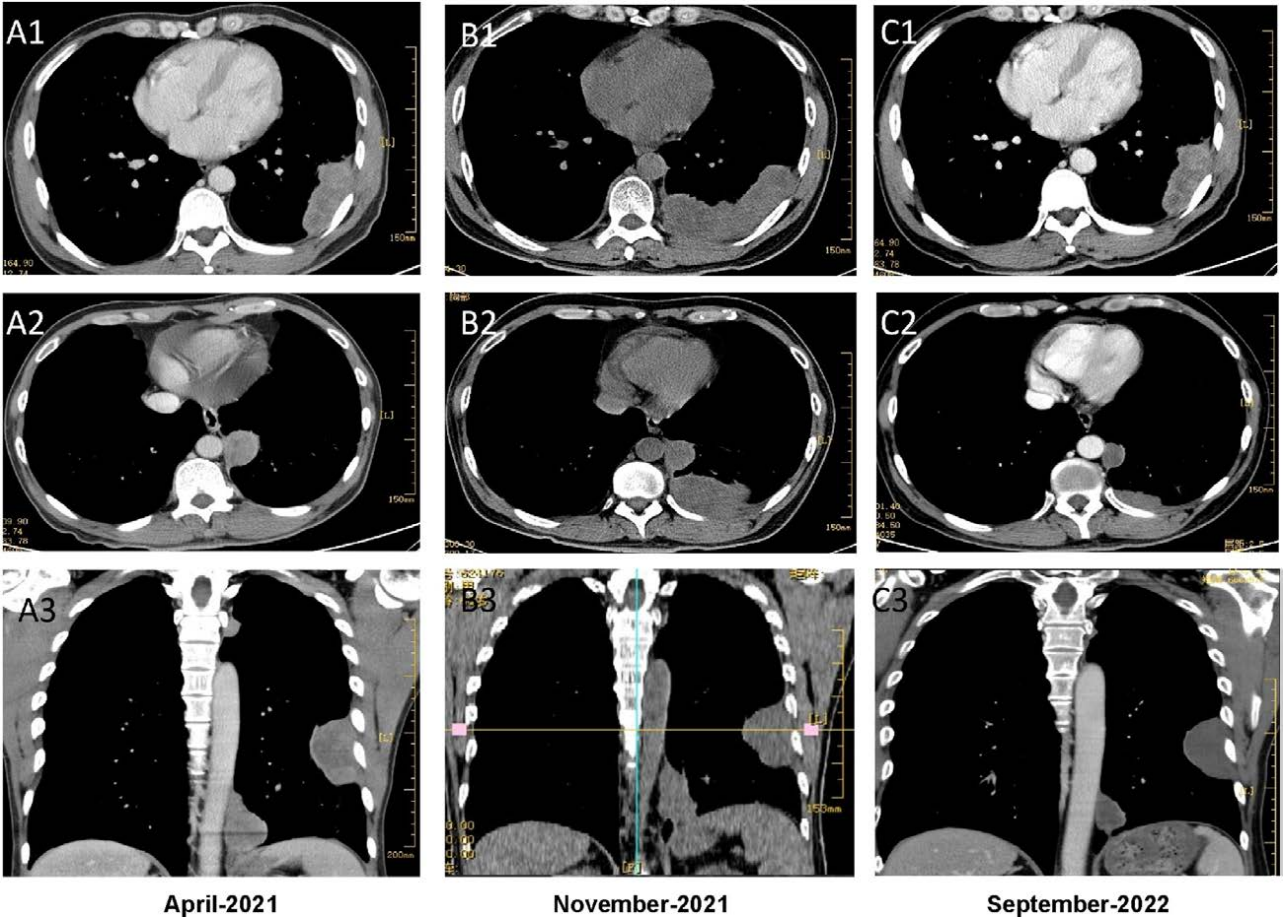
CT changes around treatment. A1–3, before treatment. B1–3, progression after first-line treatment. C1–3, after 10 months of treatment with second-line tirelizumab combined with arrotinib, the lesions were shrunk with reduced solid components.

**Figure 2.**
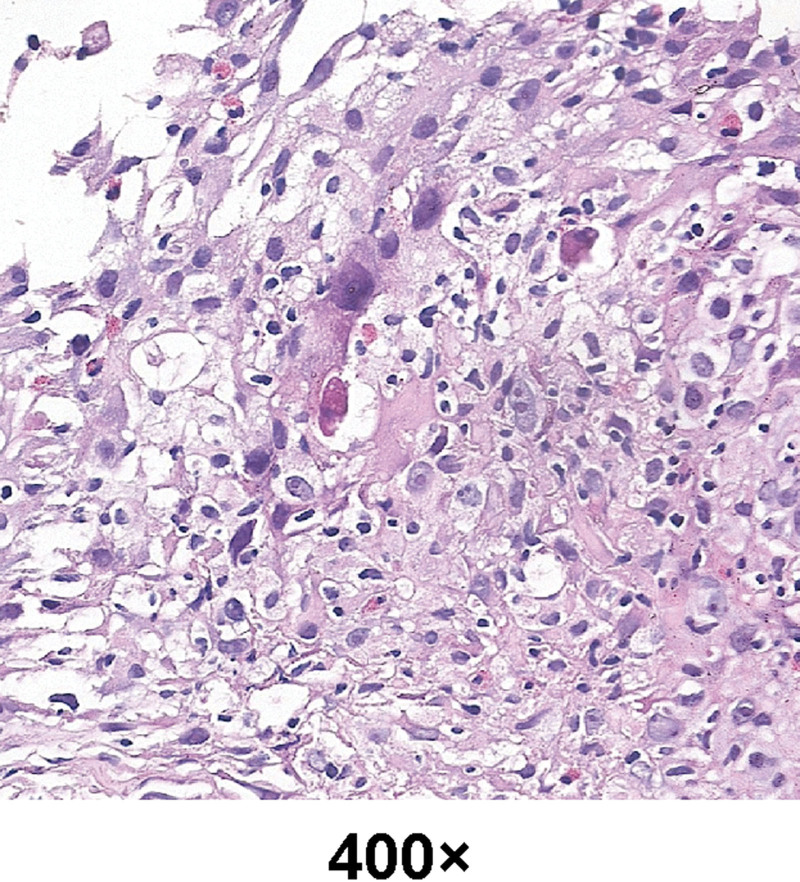
Pathological results: HE dyed.

The patient was a 59-year-old male employee of a professional construction company. On April 30, 2021, he complained of “cough, expectoration of sputum for 1 month, and shortness of breath for 2 days” and underwent enhanced chest CT. As shown in Figure 1, A1–3, multiple masses and nodules of sizes up to 76 mm*32 mm were discovered in left pleura which were considered as pleural tumors. Then ultrasound-guided biopsy of left pleural mass was performed, followed by pathology examination (Fig. 2), which classified the lesion as malignant pleural mesothelioma, epithelioid subtype. The illness was eventually evaluated as T4N0M0, stage IIIB that matched no indication for surgery by multidisciplinary consultation. The patient was thus prescribed with the first-line treatment, namely 860 mg pemetrexed disodium and 130 mg nedaplatin plus 500 mg bevacizumab at day one of every 21 days as a course and administrated for 6 courses. After 6 cycles, the prescription was change to pemetrexed plus bevacizumab for 2 cycles. In the meantime, the patient reflected exacerbated pain and increased demand for pain-relieving drugs. Moreover, from the third month of the regimen, the patient suffered repeated anemia and received several red blood cell transfusions to correct anemia.

Meanwhile, the lesions progressed slowly but significantly accelerated at 6 months of treatment as manifested by chest CT (Fig. 1, B1–3). With the consent of the patient and his family, a second-line regimen was applied. Due to economic considerations and drug availability, dual immunotherapy regimens such as anti-CTLA-4 were not adopted to their second-line alternatives. In addition, current studies suggest that the benefit of single-agent immunotherapy in second-line application is limited, and the case has been found negative for PD-L1 expression. Therefore, the second-line scheme was comprised of PD-1 inhibitor and small molecule anti-angiogenesis drugs, namely 200 mg tislelizumab at day one plus 10mg anlotinib (qd for 2 weeks, 1 week off) for 21 days as a cycle. Approximately 2 cycles of the second-line treatment achieved gradual alleviation of his chest pain and inclined requirement for analgesic drugs, as well as improved anemia and no need for blood transfusion. After 4 cycles of the medication, many nodules and masses in the left pleura shrank as demonstrated by CT scan. The treatment has been continued for a total of 10 months and his condition of remission were maintained (Fig. 1, C1–3).

## 3. Discussion

MPM is a rare but highly fatal disease that closely related to asbestos exposure but often attacks after 20 to 40 years of the exposure.^[1,2]^ Most MPM patients have progressed to late stage at diagnosis, consequently confronted with treatment obstacles and unfavorable curative effect, leading to median OS of about 1 year, 5-year survival rate around 10% and even smaller rate of cured cases.^[2]^

Among the main interventions for MPM that include surgery and radiation therapy, systemic therapy is the only treatment able to improve overall survival as reported by randomized clinical trials^[3,4]^ despite of its limited efficacy. The recommended first-line chemotherapy regimen is pemetrexed plus cisplatin, with a median OS of about 12 months and mPFS of 5.7 months,^[4]^ but only 35% to 41% of patients respond to first-line therapy. Results of IFCT-GFPC-0701 MAPS demonstrated that the combination of bevacizumab can prolong median OS by 2.7 months and thus pemetrexed, cisplatin plus bevacizumab regimen is incorporated in first-line recommendations.^[5]^ However, in this patient, lesions continued to progress slowly and the clinical symptoms deteriorated despite the combined regimen. The progression significantly exacerbated at 6 months of administration.

Upon the resistance of MPM to first-line treatments, there is currently few standard second-line recommendations in most guidelines, including the NCCN guidelines. The second-line regimens commonly used in clinical practice are single-agent chemotherapy, such as vinorelbine, gemcitabine, etc, but only obtain mPFS of about 3 months.^[6]^ CKIs have manifested its anti-tumor effects in a variety of malignant tumors. In unresectable MPM, dual immunotherapy has achieved remarkable efficacy in the first-line application.^[4]^ Piling researches on second-line immunotherapy, despite small-scale and single-arm designs, investigated the benefit of keytruda, nivolumab, ipilimumab and so on, which only attain unsatisfactory mPFS less than 6 months.^[7–10]^

Both basic and clinical studies found that abnormal tumor microenvironment has a negative impact on the efficacy of PD-1/PD-L1 inhibitors.^[11]^ Along MPM carcinogenesis, chronic inflammatory reaction to asbestos fibers causes immunosuppressed tumor microenvironment.^[12]^ Previous studies have confirmed that anti-angiogenesis therapy can directly or indirectly alleviate the immunosuppressive state by changing the tumor microenvironment,^[13,14]^ while immunotherapy can also facilitate the normalization of tumor vasculature,^[15]^ highlighting the synergistic effect of immunotherapy and anti-angiogenesis agents in anti-tumor strategies, which has been validated in in a variety of tumors including non-small cell lung cancer, renal cell carcinoma and so forth.^[16,17]^ In particular, combination of arotinib and CKIs has proved its safety and significant advancement of efficacy in case reports and clinical researches.^[18,19]^ However, few researches focus on its application in MPM. In clinical practice, our case continued to progress after chemotherapy combined with bevacizumab. Given that he was negative for PD-L1 expression, we ruled out single drug immunotherapy when modifying treatment scheme. With the consent of the patient and his family, we chose immunotherapy combined with anti-angiogenesis drugs. As bevacizumab failed in this patient, we selected arotinib in the second line, which is a small molecule multi target anti-angiogenesis drug that inhibits VEGFR 1/2/3 (vascular endothelial growth factor receptor 1/2/3) and other major tyrosine kinase receptors (such as FGFR1-4, PDGFR α/β, C-Kit, and FLT3) to potently block tumor angiogenesis and suppress proliferation. Second-line treatment with arotinib and tirelizumab significantly relieved his clinical symptoms and imaging examination also verify the improvement. At present, his PFS of second-line treatment has sustained 10 months, presenting satisfactory responsiveness that markedly exceeds the existing second-line scheme in contemporary researches.

## 4. Conclusion

Here we report a case that firstly demonstrated the efficacy of tislelizumab combined with anlotinib in the second-line treatment of MPM. Thus, immunotherapy combined with small-molecule multi-target anti-angiogenic therapy may be alternative for the second-line treatment of MPM.

## Author contributions

**Conceptualization:** Dandan Zhang, Weihong Guo.

**Data curation:** Weihong Guo, Jianping Liang.

**Formal analysis:** Dandan Zhang.

**Investigation:** Jianping Liang.

**Methodology:** Yanhua Lv, Jianping Liang.

**Software:** Xikun Huang.

**Writing – original draft:** Dandan Zhang, Weihong Guo.

**Writing – review & editing:** Dandan Zhang, Weihong Guo.
